# Functional Coupling of Calcium-Sensing Receptor and Polycystin-2 in Renal Epithelial Cells: Physiological Role and Potential Therapeutic Target in Polycystic Kidney Disease

**DOI:** 10.3390/ijms262412004

**Published:** 2025-12-13

**Authors:** Annarita Di Mise, Angela Ferrulli, Mariangela Centrone, Maria Venneri, Marianna Ranieri, Grazia Tamma, Rosa Caroppo, Giovanna Valenti

**Affiliations:** 1 Department of Biosciences, Biotechnologies and Environment, University of Bari, 70125 Bari, Italy; angela.ferrulli@icsmaugeri.it (A.F.); mariangela.centrone@uniba.it (M.C.); maria.venneri@icsmaugeri.it (M.V.); marianna.ranieri@uniba.it (M.R.); grazia.tamma@uniba.it (G.T.); rosa.caroppo@uniba.it (R.C.); 2 Department of Biotechnology and Biosciences, University of Milano-Bicocca, 20126 Milan, Italy; 3 Istituti Clinici Scientifici Maugeri, IRCCS, Institute of Bari, 70124 Bari, Italy

**Keywords:** CaSR, PC2, PC1, ADPKD, calcimimetics

## Abstract

Autosomal Dominant Polycystic Kidney Disease (ADPKD) is caused by mutations in *PKD1* or *PKD2* genes, encoding polycystin-1 (PC1) or polycystin-2 (PC2), respectively, characterized by excessive cell proliferation and fluid secretion, resulting in renal cyst formation and growth. PC1 and PC2 form a complex localized on the plasma membrane, endoplasmic reticulum, and primary cilia. PC2 is a non-selective cation channel which, in renal epithelial cells, contributes to calcium transport and signaling. It has been previously shown in renal cells that high external calcium increases whole-cell currents likely mediated by PC2. In this study, we explored the possibility that the Calcium Sensing Receptor (CaSR) is involved in the functional regulation of PC2. To test this hypothesis, human conditionally immortalized Proximal Tubular Epithelial cells, isolated from urine sediments, wt or with stably downregulated *PKD1* (PC1KD) or *PKD2* (PC2KD) were used. Interestingly, CaSR and PC2 co-immunoprecipitated and Proximity Ligation Assay demonstrated a direct physical interaction at endogenous protein levels. Membrane potential measurements demonstrated that selective CaSR activation, elicited by the calcimimetic R568, caused plasma membrane depolarization, consistent with the modulation of PC2-mediated cation currents, which was significantly lower in PC2KD with respect to wt and PC1KD cells. To conclude, this study provides evidence for a functional coupling of CaSR and PC2, which might be relevant for therapeutic strategies to correct dysregulations occurring in ADPKD.

## 1. Introduction

Polycystin 2 (PC2), encoded by the *PKD2* gene, is a member of the superfamily of TRP nonselective cation channels and plays a pivotal role in cellular calcium and cation homeostasis [1]. PC2 is ubiquitously expressed; in particular, it is located on the plasma membrane, endoplasmic reticulum, and primary cilia [2]. Mutations in *PKD2* contribute to approximately 15–20% of Autosomal Dominant Polycystic Kidney Disease (ADPKD) cases [3], the most common inherited monogenic renal disease worldwide, characterized by clusters of fluid-filled cysts in both kidneys, associated with a gradual decline in renal function [4]. The 85% of ADPKD clinical cases are caused by mutations in the *PKD1* gene, encoding polycystin 1 (PC1) [4].

PC2 is part of the TRP channel family and acts as a calcium-permeable cation channel, either alone as a homomeric tetramer or in complex with PC1 and other TRP channels [5,6]. Its primary function relies on the maintenance of regular calcium homeostasis, pivotal for preventing the advancement of ADPKD. Therefore, PC2-mediated calcium signaling dysregulation is believed to significantly contribute to cyst formation [7,8].

In the primary cilium of renal epithelial cells, PC1 and PC2 contribute to fluid-flow sensation and function as mechanosensitive protein in response to flow and pressure across the cell membrane to transmit calcium signals [9,10,11,12]. A recent study showed that PC2 forms a functional ion channel in primary cilia, without PC1, which preferentially conducts the monovalents K^+^ and Na^+^, over divalent Ca^2+^ ions [13]. On the plasma membrane, calcium influx through a functional PC2 seems to be essential to control the delivery of Ca^2+^ to the cytoplasm [14]. However, little is known about the endogenous PC2 expression on the plasma membrane, as well as the putative calcium-dependent mechanisms that may regulate its function. Recently, it has been demonstrated that LLC-PK1 cells respond to changes in external Ca^2+^ with the activation and redistribution of PC2 to the plasma membrane via the extracellular calcium sensing receptor, CaSR [15]. CaSR is a G-protein-coupled receptor which senses changes in extracellular calcium concentrations and regulates parathyroid hormone secretion and renal tubular calcium reabsorption to maintain serum calcium levels within the normal range [16]. We already showed that conditionally immortalized human Proximal Tubular Epithelial Cells (PTEC) isolated from urine of a healthy subject or of a PKD1 patient, or upon stable downregulation of *PKD1*, express a functional CaSR [17]. The specific activation of CaSR elicited by its allosteric modulator, the calcimimetic R568, induced an increase in cytosolic Ca^2+^, a decrease in intracellular cAMP level and mTOR activity, and restored the altered mitochondrial function, all events known to promote cyst formation in ADPKD [18,19].

In the present study, we show for the first time the physical interaction between PC2 and CaSR and the potential modulation of PC2 cation channel activity by calcimimetic.

## 2. Results

### 2.1. PC2 and CaSR Expression in PTEC

**Figure 1:**
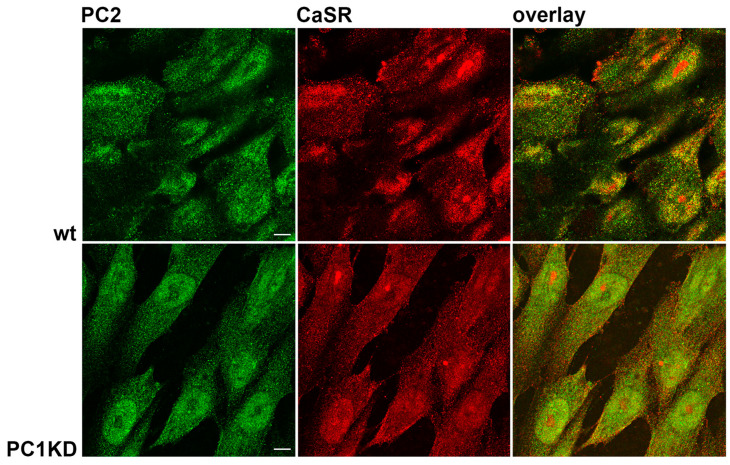
Immunofluorescence localization of endogenous CaSR and PC2 in PTEC wt and PC1KD. Overlay of the two immunostaining pictures demonstrates that the two proteins co-localize in both cell lines (scale bars: 10 μm).

Immunofluorescence staining revealed the endogenous CaSR and PC2 expression in PTEC stably knocked down for polycystin-1 (PC1KD) and in the wild type clone (wt) (Figure 1). Both cell types showed a diffuse co-immunolocalization of the two proteins, as shown by the overlay images.

### 2.2. PC2 and CaSR Co-Immunoprecipitation in PTEC and Mouse

**Figure 2:**
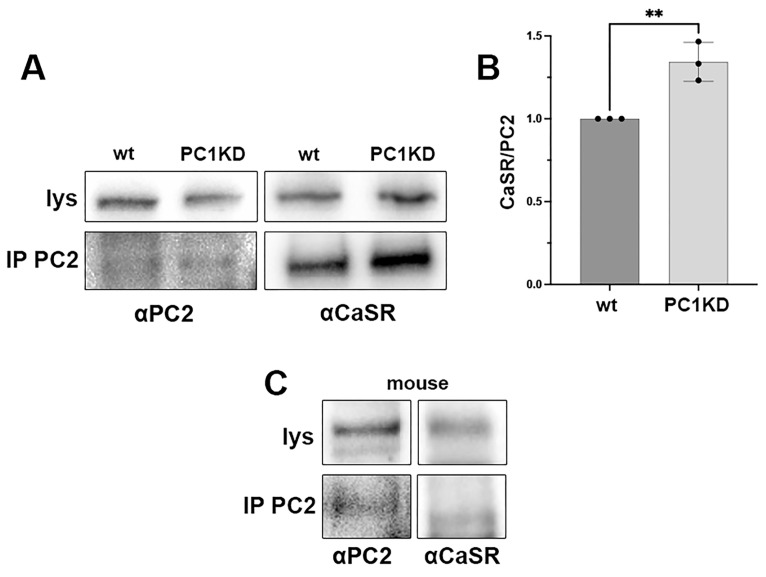
PC2 and CaSR co-immunoprecipitation. (**A**) PTEC wt and PC1KD homogenates were subjected to immunoprecipitation with specific antibodies against PC2 (IP PC2). Immunoprecipitates were probed with anti-CaSR antibodies. CaSR and PC2 expression in wt and PC1KD lysates is shown as positive control. (**B**) Densitometric analysis of CaSR co-immunoprecipitated with PC2 in PTEC wt and PC1KD show a higher expression of CaSR in PC1KD compared with wt (n = 3, ** *p* = 0.007, Student’s *t*-test). (**C**) Co-immunoprecipation of CaSR and PC2 performed in mouse homogenates.

Specific anti-PC2 antibodies were used for immunoprecipitation from PTEC wt and PC1KD lysates. Immunoprecipitates were next probed with anti-CaSR antibodies, revealing positive CaSR bands in both cell lines (Figure 2A). The two proteins were demonstrated to co-immunoprecipitate also from mouse kidney homogenates, thus suggesting a physiological interaction in the native tissue (Figure 2B). Interestingly, the amount of CaSR immunoprecipitated with PC2 was significantly higher in PC1KD cells with respect to wt (wt = 1, PKD1 = 1.34 ± 0.07, *p* = 0.007), highlighting an increased functional interaction of the receptor to PC2 when PC1 is knocked down (Figure 2C).

### 2.3. PC2 and CaSR Interaction

**Figure 3:**
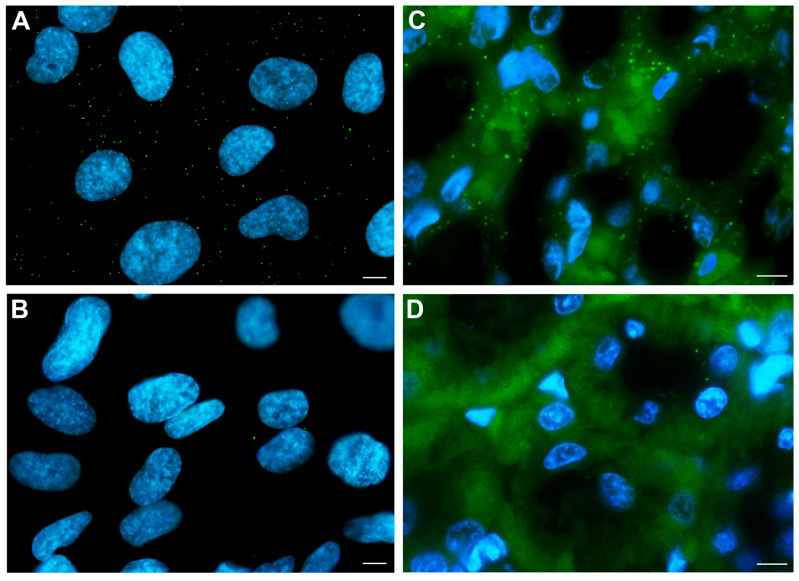
Proximity ligation assay images showing CaSR and PC2 in situ interaction (at distances < 40 nm) in a confluent monolayer of PTEC wt (**A**) or in a rat kidney section (**C**). Green bright PLA dots, indicating actual interaction between CaSR and PC2, are shown. Nuclei are stained in blue. Images in (**B**) (PTEC wt) and (**D**) (rat kidney section) show negative controls without primary antibodies (scale bars: 10 μm). PC2 antibody-deficient controls are showed in Figure S1.

To evaluate the actual physical interaction between CaSR and PC2 suggested by the co-IP data, Proximity Ligation Assay (PLA) experiments were performed, allowing the detection of protein–protein interaction at endogenous protein levels [20,21]. CaSR and PC2 in situ interaction (at distances < 40 nm) in confluent monolayers of PTEC wt and rat kidney section were observed both in cells (Figure 3A) and in the native tissue (Figure 3C). 

### 2.4. Membrane Potential Measurements

To evaluate whether the physical interaction between CaSR and PC2 had a functional role in PC2 cation channel activity, PTEC wt and PC1KD were loaded with bis-oxonol fluorophore, DiBAC_4_(3), a membrane potential-sensitive probe, which allows for the measurement of the relative cellular membrane potential. The effect of the specific activation of CaSR was evaluated by stimulating cells with CaSR allosteric modulator, the calcimimetic R568 in both cell lines.

**Figure 4:**
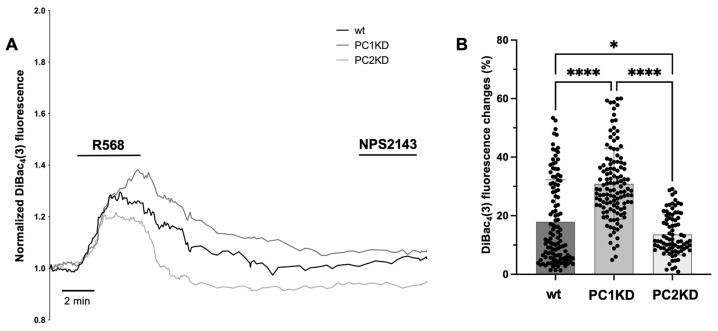
Membrane potential measurements in PTEC wt, PC1KD and PC2KD performed with the potential-sensitive probe DiBAC_4_(3). (**A**) Representative traces of membrane potential measurements after specific CaSR stimulation, elicited by the calcimimetic R568, followed by CaSR inhibition with the calcilytic NPS2143. (**B**) Data are expressed as the percentage change in fluorescence from basal, registered before the stimulus, to the peak. Histogram shows a significant increase in the depolarization rate in PC1KD cells compared with wt and PC2KD, after R568 treatment. PC2KD cells showed a significantly lower depolarization rate with respect to wt as well. Data were analyzed with One-way ANOVA followed by Tukey’s multiple comparisons test and are expressed as means ± SEM (**** *p* < 0.0001, * *p* < 0.05).

To validate the cellular response to depolarizing or hyperpolarizing stimuli, cells were perfused with 20 mM K^+^ or 100 μM ATP solutions, which induced an increase in fluorescence, corresponding to a depolarization, or a decrease in fluorescence, reporting a hyperpolarization, respectively (Figure S2). Specific CaSR activation using R568 (10 μM), led to membrane potential depolarization, which was significantly lower in wt cells (17.9 ± 1.3%, n = 117) with respect to PC1KD (30.9 ± 1.1%, n = 124, *p* < 0.0001 vs. wt) (Figure 4A, and relative statistical analysis in Figure 4B).

The depolarization was evaluated by single-cell epifluorescence imaging and expressed as percentage of the fluorescence increment from the basal value, registered before the stimulus, to the peak. Interestingly, PC2KD cells showed an even lower depolarization with respect to wt or PC1KD cells (13.6 ± 0.8%, n = 84, *p* < 0.05 vs. wt, *p* < 0.0001 vs. PC1KD) (Figure 4B). The specific role of CaSR activation in inducing plasma membrane depolarization was confirmed by control experiments stimulating PTEC wt, PC1KD or PC2KD with the specific CaSR antagonist, the calcilytic NPS2143, which had no effect on membrane potential (Figure 4A).

**Figure 5:**
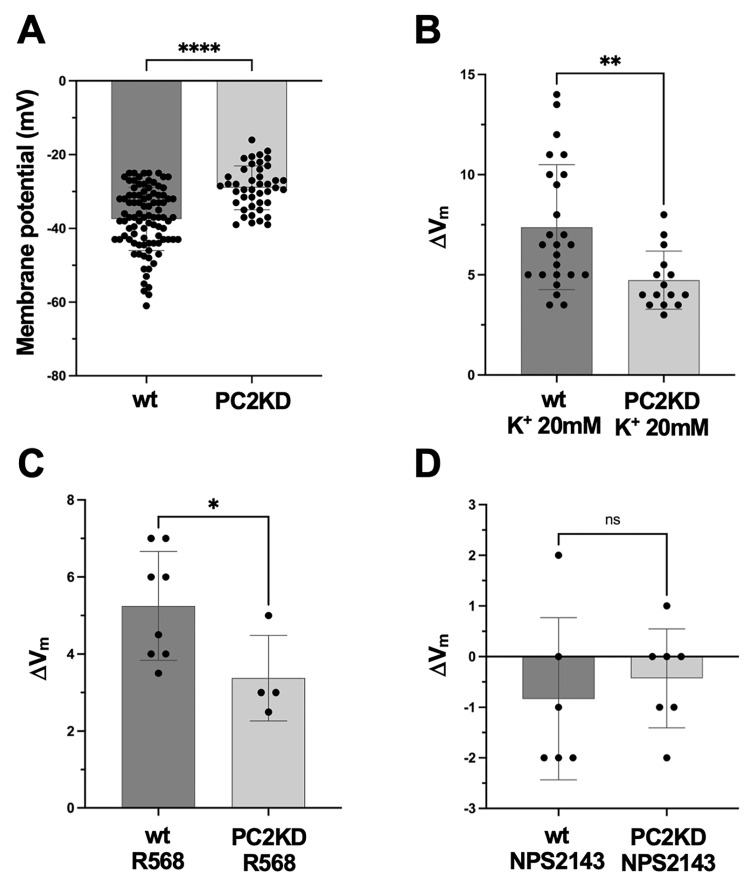
Microelectrodes measurements in PTEC wt and PC2KD. (**A**) Resting membrane potential measurements. Histogram shows a significantly higher membrane potential in PTEC wt compared with PC2KD (**** *p* < 0.0001). (**B**) Membrane potential changes (∆V_m_) in response to increased extracellular K^+^ concentration. Histogram shows a significantly higher membrane depolarization in wt cells with respect to PC2KD (** *p* < 0.005). (**C**) Effect of CaSR selective activation elicited by R568 on membrane potential. Histogram shows a statistically higher membrane depolarization in wt cells (* *p* < 0.05). (**D**) The specific inhibition of CaSR with its antagonist NPS2143 did not induce any significant change in the membrane potential of PTEC wt and PC2KD (n.s.: not significant). Data were analyzed by Student’s *t*-test and are expressed as means ± SEM.

The functional interaction between CaSR and PC2 was further validated by measuring the membrane potential (V_m_) of PTEC wt or PC2KD with intracellular microelectrodes, to quantify the changes in V_m_ observed in response to CaSR activation. A significantly different resting membrane potential was observed in wt and PC2KD cells. Specifically, the average resting V_m_ recorded in wt and PC2KD cells was –37.5 ± 0.9 mV (n = 94) and –29.0 ± 0.9 mV (n = 43), respectively (*p* < 0.0001) (Figure 5A). During the V_m_ recording, the microelectrode sensitivity was assessed by increasing K^+^ concentration in the extracellular medium from 4 to 20 mM. As expected, this resulted in membrane depolarization which was significantly higher in wt cells compared to PC2KD (wt: ∆Vm = 7.4 ± 0.6 mV, n = 25; PC2KD: ∆V_m_ = 4.7 ± 0.4 mV, n = 15, *p* < 0.005) (Figure 5B). These changes were promptly reversible upon returning to basal conditions, thus confirming the reliability of the microelectrode measurements. The observation that PC2KD cells exhibited a smaller depolarization in response to the increased extracellular K^+^ concentration suggests that PC2 is involved in the basal K^+^ permeability. This could also explain the lower resting V_m_ recorded in these cells (Figure 5A). Taken together, these findings indicate a role for PC2 in the basal efflux of K^+^, contributing to the regulation of the resting membrane potential in PTEC.

The effects of CaSR activation on V_m_ in both cell lines were next investigated. In line with the data obtained with DiBAC_4_(3), the addition of R568 (10 µM) to the bathing solution caused a depolarization of the cell membrane which was significantly lower in PC2KD cells compared to wt (wt: ∆V_m_ = 5.3 ± 0.5 mV, n = 8; PCKD2: ∆V_m_ = 3.4 ± 0.6 mV, n = 4, *p* < 0.05) (Figure 5C). The specific involvement of CaSR was then confirmed by using the calcilytic NPS2143. As showed in Figure 5D, CaSR antagonist did not evoke any significant V_m_ change in both cell lines (wt: ∆V_m_ = −0.8 ± 0.7 mV, n = 6; PC2KD: ∆V_m_= −0.4 ± 0.4 mV, n = 7). The lack of significant V_m_ change upon NPS2143 addition confirmed the specificity of CaSR in mediating the depolarizing effects observed with R568.

## 3. Discussion

The present study shows the interaction and functional interplay between polycystin-2 (PC2) and the calcium sensing receptor (CaSR), in conditionally immortalized human Proximal Tubular Epithelial Cells (PTEC), wt or downregulated for *PKD1* (PC1KD).

The endogenous expression of PC2 and CaSR in both PTEC wt and PC1KD was assessed via immunofluorescence staining. Interestingly, both cell lines exhibited diffuse co-localization of the two proteins, which was further demonstrated by co-immunoprecipitation experiments. Of note, considering the same amount of immunoprecipitated PC2 in both cell lines, the rate of co-immunoprecipitated CaSR was significantly higher in PC1KD cells compared with PTEC wt, indicating that the interaction between CaSR and PC2 can be modulated in response to the expression or down-regulation of PC1 (polycystin-1). The physical interaction between CaSR and PC2 was further proved by Proximity Ligation Assay (PLA). Notably, we showed the in situ interaction between CaSR and PC2 in PTEC and rat kidney sections, corroborating the co-localization of the two proteins and demonstrating their physical relationship.

To better understand the functional implications of CaSR and PC2 interaction, membrane potential measurements were performed using the DiBAC_4_(3) probe, which is sensitive to changes in cellular membrane potential. The selective activation of CaSR by the calcimimetic R568 led to membrane depolarization in PTEC wt, which was significantly higher in PC1KD cells, indicating a higher calcium current mediated response to CaSR activation when PC1 is silenced. This suggests that the interaction between CaSR and PC2 is enhanced when PC1 is downregulated and is consistent with a higher calcium influx through PC2, which on the plasma membrane presents a preferentially selective permeability to Ca^2+^ over Na^2+^ and K^+^ [22,23]. The contribution of plasma membrane PC2 to the CaSR-regulated depolarization was confirmed by membrane potential measurements performed in PC2KD cells, downregulated for *PKD2*. In this cell line, the specific CaSR activation, elicited by R568, induced a significantly lower plasma membrane depolarization with respect to PTEC wt and PC1KD. This further highlights the critical role of PC2 in regulating membrane potential in response to CaSR activation, whose selective involvement was further confirmed by stimulating cells with the specific CaSR antagonist, NPS2143, which did not elicit any change in the membrane potential. The functional interaction between CaSR and PC2 was further demonstrated by microelectrodes measurements which suggested that PC2 is crucial for CaSR-mediated changes in membrane potential. CaSR stimulation, in fact, induced depolarization of the V_m_ in both PTEC wt and PC2KD. However, the magnitude of the depolarization was significantly lower in PC2KD cells compared to wt, indicating that PC2 plays a key role in mediating the effects on ion permeability elicited by CaSR activation.

These results highlight the role of CaSR in modulating membrane potential in PTEC, with PC2 enhancing this effect. Further studies are required to fully understand the underlying mechanisms of this interaction and its implications for cellular function. The findings from this study provide significant insights into the molecular interplay between PC2 and CaSR in PTEC silenced for PC1, confirming that PC2 can function as an independent ion channel in the absence of PC1, as previously demonstrated in the primary cilia of inner medullary collecting duct epithelial cells from *Pkd1* KO mice [13]. The increased interaction between these proteins in the absence of PC1, and the CaSR-modulated PC2-associated calcium currents may suggest a compensatory mechanism to ameliorate the altered calcium signaling, which represents one of the molecular dysregulations underlying Autosomal Dominant Polycystic Kidney Disease (ADPKD).

On the other hand, the functional interplay between CaSR and PC2 further supports the idea that CaSR may represent a therapeutic target in ADPKD as suggested by our previous studies as well as by findings by other investigators [24], showing the beneficial effects of CaSR in reducing cyst enlargements in human ADPKD cells and renal cyst index in mouse models of ADPKD. In addition, interesting data in hemodialysis patients with ADPKD demonstrated that the rate of increase in total kidney volume in these patients treated with the calcimimetic cinacalcet was significantly suppressed [25].

## 4. Materials and Methods

### 4.1. Materials

All chemicals were obtained from Sigma-Aldrich/Merck (Merck KGaA, Darmstadt, Germany). CaSR positive allosteric modulator, R568 hydrochloride, and CaSR antagonist, NPS2143, were purchased from Tocris (Bio-Techne Srl, Milan, Italy). DiBAC_4_(3) (Bis-(1,3-Dibutylbarbituric Acid) Trimethine Oxonol) was obtained from Invitrogen™ (Thermo Fisher Scientific, Waltham, MA, USA). Cell culture media were from Biowest (Biowest, Nuaillé, France). Fetal bovine serum (FBS; cat no. 10270106) was from GIBCO™ (Thermo Fisher Scientific, Waltham, MA, USA). Clarity™ Western ECL substrates (cat. No #170-5061) and Clarity Max™ Western ECL Substrate (cat. No #1705062) were from Bio-Rad (Bio-Rad Laboratories, Hercules, CA, USA).

### 4.2. Antibodies

Monoclonal CaSR antibodies recognizing amino-acid 15–29 at the extracellular N-terminus were from Sigma-Aldrich (Milan, Italy) (catalogue no. C0493). Rabbit anti-Polycystin-2 was from Millipore (Merk, Burlington, MA, USA) (catalogue no. AB9088). For immunoprecipitation, anti-Polycystin-2 antibodies were from Santa Cruz Biotechnologies (Dallas, TX, USA). Secondary goat anti-rabbit (catalogue no. A0545) or anti-mouse (catalogue no. A9044) horseradish peroxidase-coupled antibodies were obtained from Santa Cruz Biotechnologies (Dallas, TX, USA). Secondary goat anti-rabbit Alexa Fluor 488 (catalogue no. A-11008) and goat anti-mouse Alexa Fluor 555 (catalogue no. A-21422) antibodies were from Molecular Probes (Eugene, OR, USA).

### 4.3. Cell Culture

Human conditionally immortalized Proximal Tubular Epithelial Cells (PTEC) were generated by Wilmer and colleagues [26]. Primary cells were cultured by collecting mid-stream urine and immortalized as previously described [26]. Stable knocked down PTEC for polycystin-1 (PC1KD) or polycystin-2 (PC2KD) were obtained by transducing a cloned PTEC line (PTEC wt) of a healthy individual by using lentiviral vectors encoding miR-shRNA directed against PC1 (pCHMWS Bsd 2xmiRNA PKD1) or PC2 (pCHMWS Bsd 2xmiRNA PKD2) [27]. Transduced cells were selected adding 10 g/mL blasticidin to the culture medium: DMEM Ham’s F12 medium supplemented with 10% fetal bovine serum (FBS), 100 IU/mL penicillin, 100 mg/mL streptomycin, ITS (5 μg/mL insulin, 5 μg/mL transferrin and 5 ng/mL selenium), 36 ng/mL hydrocortisone, 10 ng/mL epidermal growth factor (EGF) and 40 pg/mL triiodothyronine. Experiments were performed prior cellular maturation for 11 days at 37 °C.

### 4.4. Immunofluorescence Microscopy

Immunofluorescence localization of CaSR and PC2 in polarized PTEC was performed as previously described [17,28]. Cells were incubated with antibodies diluted in blocking solution containing 2% (*w*/*v*) bovine serum albumin (BSA) and 0.1% (*v*/*v*) tween-20 in HBSS against CaSR and PC2 at 4 °C overnight. After incubation with secondary goat anti-mouse Alexa 488 and secondary goat anti-rabbit Alexa 555 antibodies, coverslips were mounted on glass slides with Mowiol. Images were obtained with a confocal microscope Leica TCS SP2 (Leica Microsystems, Heerbrugg, Switzerland).

### 4.5. Immunoprecipitation

Immunoprecipitation experiments were performed as previously described [17,29]. Briefly, PTEC were lysed with a buffer containing 1% Triton X-100, 150 mM NaCl, and 25 mM Hepes (pH 7.4), in the presence of protease inhibitors (1 mM PMSF, 2 mg/mL leupeptin, and 2 mg/mL pepstatin A). The supernatants were precleared with protein A-Sepharose solution and incubated overnight with anti-PC2 antibodies coupled to protein A-Sepharose. The immunocomplexes were washed, resuspended in Laemmli buffer, and subjected to immunoblotting using CaSR and PC2 antibodies.

### 4.6. Gel Electrophoresis and Immunoblotting

PTEC lysates were separated on 7.5% Bis-Tris acrylamide gels under reducing conditions, as described in [30,31]. Protein bands were electrophoretically transferred onto Immobilon-P membranes (Millipore) for Western blot analysis, blocked in EveryBlot blocking buffer (Bio-Rad Laboratories, Milan, Italy), and incubated with primary antibodies overnight. Immunoreactive bands were detected with secondary antibodies conjugated to horseradish peroxidase. Membranes were developed using Clarity™ western and Clarity™ Max western ECL substrate with Chemidoc System (Bio-Rad Laboratories, Milan, Italy).

For the quantification of CaSR co-immunoprecipitated with PC2, the immunoprecipitated (IP) amounts of CaSR and PC2 were first normalized to their respective immunoglobulin G (IgG) controls, then normalized to the corresponding protein abundances in the lysates. The amount of IP CaSR was subsequently expressed as the ratio CaSR/PC2.

### 4.7. Proximity Ligation Assay (PLA)

PLA was performed using the DuoLink In Situ Detection Reagents Green kit (DUO92014, Sigma-Aldrich), according to the supplier’s recommendations. Briefly, cells seeded on 12 mm glass coverslips were fixed for 20 min with 4% paraformaldehyde in PBS. Samples were permeabilized in SDS 0.1% PBS for 5 min, washed, blocked for 1 h at 37 °C and incubated overnight at 4 °C with mouse anti-CaSR and rabbit anti-PC2 antibodies. Subsequently, samples were washed and incubated 1 h at 37 °C with the secondary antibodies, DuoLink In Situ PLA Probe Anti-Mouse PLUS (DUO92001, Sigma) and DuoLink In Situ PLA Probe Anti-Rabbit MINUS (DUO92005, Sigma). The following ligation and amplification steps were performed according to the manufacturer’s instructions. The PLA probes that bind to the constant regions of the primary antibodies contain a unique DNA strand. If the proteins of interest interact with each other, the DNA probes hybridize to form circular DNA, which is then amplified and detected by fluorescently labeled complementary oligonucleotide probes.

Images were obtained with a confocal microscope Leica TCS SP5 (Leica Microsystems, Mannheim, Germany). The PLA signals, indicating interaction between CaSR and PC2, appear as distinct fluorescent dots distributed throughout the cells.

### 4.8. Fluorescence-Based Membrane Potential Measurements

The plasma membrane relative potential was measured by using the slow-response, potential sensitive, bis-oxonol fluorophore, DiBAC_4_(3) (B-438). Briefly, cells were grown on 25 mm glass coverslips at 37 °C for 11 days and then loaded with 8 μM DiBAC_4_(3) for 45 min at 37 °C in Ringer’s solution, containing 120 mM NaCl, 4 mM KCl, 15 mM NaHCO_3_, 1 mM MgCl_2_, 15 mM Hepes, 0.5 mM NaH_2_PO_4_, 10 mM Glucose, 1 mM CaCl_2_, 0.5 mM Na_2_HPO_4_, 0.4 mM MgSO_4_, pH 7.4. The coverslips with dye-loaded cells were mounted in a perfusion chamber (FCS2 Closed Chamber System, BIOPTECHS, Butler, PA, USA) and measurements were performed using an inverted microscope (Nikon Eclipse TE2000-S microscope—Nikon Europe B.V., Amstelveen, The Netherlands), equipped for single-cell fluorescence measurements and imaging analysis. The anionic fluorophore DiBAC_4_(3) enters the cell and binds to intracellular proteins and lipid membranes, resulting in enhanced fluorescence (Ex/Em: 490/515 nm), which is captured by a cooled ECCD camera (CoolSNAP HQ, Photometrics, Tucson, AZ, USA). When the membrane potential becomes more positive (or depolarizes), the permeability of cells to DiBAC_4_(3) increases, resulting in an increased fluorescence. Conversely, a decrease in fluorescence signal corresponds to membrane hyperpolarization. The measurements of DiBAC_4_(3) intensity changes were carried out using Metafluor^®^ software v7.8.1.0 (Molecular Devices, LLC, San Jose, CA, USA). To validate the cellular response to a depolarizing stimulus, cells were perfused with 20 mM K^+^ buffer containing 104 mM NaCl, 20 mM KCl, 15 mM NaHCO_3_, 1 mM MgCl_2_, 15 mM Hepes, 0.5 mM NaH_2_PO_4_, 10 mM Glucose, 1 mM CaCl_2_, 0.5 mM Na_2_HPO_4_, 0.4 mM MgSO_4_, pH 7.4, in order to reduce the chemical K^+^ gradient (Figure S2B). The validation of cellular response to a hyperpolarizing stimulus was performed by stimulating cells with ATP 100 µM in Ringer’s solution Figure S2A).

### 4.9. Microelectrode Measurements of Plasma Membrane Potential

The cell membrane potential (V_m_) measurements were performed according to a protocol previously described [32]. Briefly, before recordings, glass coverslips with cells were mounted in a modified open-top perfusion chamber (Series 20, Warner Instrument Corp, Hamden, CT, USA). Individual cells were micropunctured with intracellular microelectrodes made from borosilicate glass (Hilgenberg, Malsfeld, Germany) mounted on a Leitz micromanipulator connected to a dual-channel electrometer (WPI, Sarasota, FL, USA) and to a strip-chart recorder (Kipp and Zonen, Delft, The Netherlands). A calomel half-cell filled with 2.7 mol/L KCl solution was connected via an agar/Ringer bridge to the suction well of the chamber downstream of the cells and used as reference electrode. The cells were continuously perfused with Ringer’s solution.

### 4.10. Statistical Analysis

The analyses were carried out using GraphPad Prism, version 10. One-way ANOVA followed by Tukey’s multiple comparisons test or Student’s *t*-test for unpaired data were used. All values are expressed as means ± SEM. A difference of *p* < 0.05 was considered statistically significant.

## 5. Conclusions

The present study provides novel insights on the physical and functional interaction between PC2 and CaSR, in human proximal tubular epithelial cells. It demonstrates that CaSR activation modulates PC2 cation channel activity, particularly in *PKD1*-silenced cells, where the two proteins show increased co-immunoprecipitation compared with wt, and where specific CaSR activation induces a significantly higher membrane potential depolarization. These findings suggest a compensatory mechanism for calcium homeostasis in ADPKD, which represents one of the major dysregulated pathways in this disease, supporting the CaSR as a potential therapeutic target.

## Data Availability

The original contributions presented in this study are included in the article/Supplementary Materials. Further inquiries can be directed to the corresponding author.

## Supplementary Materials

The following supporting information can be downloaded at: https://www.mdpi.com/article/10.3390/ijms262412004/s1.
