# Supplementary material for: Functional Coupling of Calcium-Sensing Receptor and Polycystin-2 in Renal Epithelial Cells: Physiological Role and Potential Therapeutic Target in Polycystic Kidney Disease
Source: Int J Mol Sci. 2025 Dec 13;26(24):12004. doi: 10.3390/ijms262412004 (PMC12732451; doi:10.3390/ijms262412004)

Supplementary figures

Figure S1. Proximity ligation assay. PC2 antibody-deficient controls in PTEC wt (A) and rat kidney section (B).

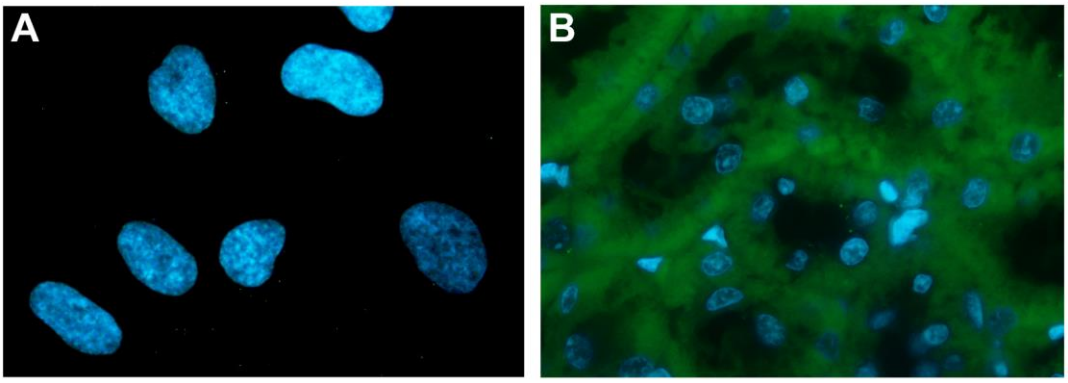

Figure S2. Representative traces of membrane potential measurements in PTEC wt, PC1KD and PC2KD, performed using the potential-sensitive probe DiBAC$_4$(3). Cells were perfused with ATP 100µM (A), used as a hyperpolarizing stimulus, resulting in a decrease in fluorescence, and with K^+ 20 mM solution (B), used as a depolarizing stimulus, inducing an increase in fluorescence.

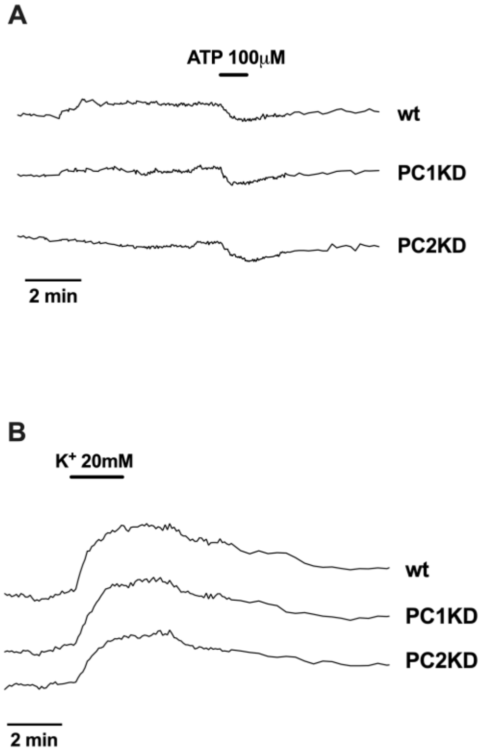

Supplement: Supplementary file 1 [file ijms-26-12004-s001.zip › ijms-3780553-supplementary.pdf]
